# Additional PD-1 inhibitor improves complete response to induction chemotherapy in locally advanced nasopharyngeal carcinoma

**DOI:** 10.3389/fimmu.2024.1415246

**Published:** 2024-06-06

**Authors:** Yi-Feng Yu, Guan-Zhong Lu, Run-Jie Wang, Yu-Kun Song, San-Gang Wu

**Affiliations:** ^1^ Department of Radiation Oncology, Xiamen Cancer Quality Control Center, Xiamen Cancer Center, Xiamen Key Laboratory of Radiation Oncology, the First Affiliated Hospital of Xiamen University, School of Medicine, Xiamen University, Xiamen, China; ^2^ Department of Radiology, the First Affiliated Hospital of Xiamen University, School of Medicine, Xiamen University, Xiamen, China

**Keywords:** nasopharyngeal carcinoma, chemotherapy, induction therapy, immunotherapy, response

## Abstract

**Purpose:**

To investigate the treatment response and toxicity of the combination of induction chemotherapy (IC) and PD-1 inhibitor in locally advanced nasopharyngeal carcinoma (LANPC).

**Methods:**

Patients with stage III–IVA NPC who received IC or IC + PD-1 inhibitor were included. The chi-square test and multivariate logistic regression analysis were used for statistical analysis.

**Results:**

A total of 225 patients were identified, including 193 (85.8%) and 32 (14.2%) who received IC alone and IC + PD-1 inhibitor, respectively. The addition of PD-1 inhibitor to IC significantly improved the tumor response than those treated with IC alone. The complete response (CR), partial response, stable disease, and progressive disease rates of 4.7% vs. 31.3%, 69.4% vs. 62.5%, 24.9% vs. 6.3%, and 1.0% vs. 0% in patients receiving IC alone and IC + PD-1 inhibitor, respectively (P<0.001). The results of the multivariate logistic regression showed that receiving PD-1 inhibitor was an independent predictor influencing the CR rate of patients (odds ratio 9.814, P<0.001). The most common toxicity by using IC and PD-1 inhibitor was hematological toxicity. In terms of non-hematological toxicity, 7 (21.9%) patients experienced thyroid dysfunction and all of them were hyperthyroidism. No grade 5 toxicities were found. In those who received IC and PD-1 inhibitor, the one-year locoregional recurrence-free survival, distant metastasis-free survival, disease-free survival, and overall survival were 100%, 96.9%, 96.9%, and 100%, respectively.

**Conclusion:**

The addition of PD-1 inhibitor to IC has promise as an effective treatment approach for LANPC. More studies are expected to provide further insights into the optimal use of this treatment strategy, paving the way for more personalized and effective treatment options for patients with LANPC.

## Introduction

Nasopharyngeal carcinoma (NPC) is a malignant tumor originating from the epithelial cells of the nasopharynx. It is relatively rare worldwide but has a higher incidence in certain regions, particularly Southeast Asia, including China (1). There were approximately 70% of patients diagnosed with locally advanced nasopharyngeal carcinoma (LANPC) (2). The optimal treatment approach for LANPC according to the current guidelines is induction chemotherapy (IC) following concurrent chemoradiotherapy (CCRT), with a 5-year overall survival (OS) rate of approximately 85% (3). However, there were approximately 20% of patients may develop disease recurrence after comprehensive treatment, especially for those with inferior response to IC (4–6). According to the second analysis from the prospective trial, those with complete response (CR) to IC had significantly lower recurrence rates and higher survival rates (5). However, the CR rate was only 2.8–11.3% after IC in several prospective studies (5, 7, 8).

Immunotherapy has emerged as a transformative treatment approach for various malignancies, including NPC (9). The rationale behind immunotherapy in NPC lies in the unique immunogenicity and immune evasion mechanisms associated with the disease. NPC is characterized by a high frequency of Epstein-Barr virus (EBV) association, which induces the upregulation of immune checkpoint proteins, such as programmed cell death ligand-1 (PD-L1), leading to immune evasion. Immunotherapeutic agents targeting these immune checkpoints, particularly anti-PD-1/PD-L1 antibodies, have demonstrated remarkable efficacy in NPC (10–12). Several phase III studies have shown that chemotherapy plus PD-1 inhibitor had a significantly higher clinical response, progression-free survival (PFS), and OS compared to those treated with chemotherapy alone (10–12). The unique epidemiology, pathogenesis, and treatment challenges associated with NPC have prompted the exploration of novel therapeutic approaches, including the integration of IC and PD-1 inhibitor in LANPC. Therefore, this study aimed to investigate the treatment response, toxicity, and short-term survival of the combination of IC and PD-1 inhibitor in LANPC.

## Materials and methods

### Patients

We retrospectively collected data from LANPC patients who were treated at our institution from January 2019 to September 2023. Patients who met the following criteria were included: 1) histologically confirmed NPC; 2) diagnosed with LANPC (stage III-IVA disease) according to the 8th edition of the American Joint Committee on Cancer staging system); 3) received induction therapy including two-three cycles of IC and PD-1 inhibitor. We excluded patients with the following criteria: 1) underwent surgical treatment to the primary nasopharyngeal tumors and/or the metastatic cervical lymph nodes; 2) had second primary cancers simultaneously diagnosed with NPC or had other malignancy before NPC diagnosis. This study was approved by the Institutional Review Board of the First Affiliated Hospital of Xiamen University and informed consent was obtained from all the patients before treatment (approval number: 2023050).

### Variables

We include the following variables in the analysis: age, gender, smoking history, alcohol history, histology, clinical stage, tumor (T) stage, nodal (N) stage, IC regimen, PD-1 inhibitor as well as the plasma EBV-DNA levels before and after the induction treatment.

### Treatment

In our institution, the IC regimens included the TPF (docetaxel 75 mg/m^2^ or nab-paclitaxel 260mg/m^2^ on day 1, cisplatin 25 mg/m^2^ on days 1–3, and 5-FU 600–750 mg/m^2^ per day as a continuous 120 hours infusion or S1 capsules 40 mg/m^2^ bid on day 1–14), TP (docetaxel 75 mg/m^2^ or nab-paclitaxel 260mg/m^2^ on day 1, cisplatin 25 mg/m^2^ on days 1–3), or GP regimens (gemcitabine 1000 mg/m^2^ on days 1 and 8, cisplatin 25 mg/m^2^ on days 1–3). We added the PD-1 inhibitor including Camrelizumab (200 mg on day 1) or Tislelizumab (200 mg on day 1) into IC during the phase of induction therapy. Pegylated recombinant human granulocyte colony-stimulating factor could be used for the prevention of bone marrow suppression.

All patients began definitive CCRT within three weeks after the completion of induction therapy. Radiotherapy was delivered using volumetric modulated arc therapy or helical tomotherapy. Target volumes were delineated following guidelines from the Chinese Society of Clinical Oncology (CSCO) and our institution (13, 14). We primarily delineated the grosstumor volume in the nasopharynx (GTVp), gross tumor volume in the neck (GTVn), high-risk clinical target volume in the neck (CTVn1) (GTVn + 3 mm, with corresponding modifications made when adjacent to important organs at risk [OARs]), high-risk clinical target volume (CTVp1) (GTVp + 5–10mm, with corresponding modifications made when adjacent to important OARs), and low-risk clinical target volume (CTV2) (CTVp1 + 5–10 mm and cervical lymph nodes, with corresponding modifications made based on the extent of tumor invasion or proximity to important OARs). The total radiation dose for GTVp, GTVn, CTVn1, CTVp1, and CTVp2 was 70.29 Gray (Gy), 70.29 Gy, 62.04 Gy, 62.04 Gy, and 56.10 Gy, respectively, delivered in 33 fractions given five times per week. GTVp and GTVn were delineated according to the tumor area after IC, while the extent of bone and paranasal sinus infiltration was delineated based on the tumor area before IC. Concurrent chemotherapy was recommended and cisplatin (80 mg/m2 given on days 1–3, every 3 weeks) or lobaplatin (30 mg/m2 on day 1, every 3 weeks) were used with a total of two cycles.

### Assessment of treatment response

The effectiveness evaluation after induction therapy was performed using the Response Evaluation Criteria in Solid Tumors (RECIST) version 1.1. Two experienced radiation oncologists (SGW) and radiologists (YKS) evaluated the changes in the imaging of the primary nasopharyngeal tumors and the metastatic cervical lymph nodes before and after induction therapy. The evaluation criteria include CR, partial response (PR), stable disease (SD), and progressive disease (PD). The overall response rate (ORR) was calculated as the sum of CR and PR.

### Assessment of treatment toxicity

Acute toxicities during induction therapy were graded according to the Common Terminology Criteria for Adverse Events version 4.0. The toxicities related to PD-1 inhibitor were also evaluated at each treatment cycle according to the guidelines (15).

### Follow-up

In this study, survival data were collected retrospectively by reviewing medical records. All patients underwent regular follow-up assessments, including monitoring EBV-DNA levels, MRI, chest CT scans, and abdominal sonography every three months for a minimum of three years. Immediate imaging examinations, such as PET/CT, were conducted for patients showing signs of disease progression. The endpoints analyzed included locoregional recurrence-free survival (LRFS), distant metastasis-free survival (DMFS), disease-free survival (DFS), and OS. LRFS was defined as the time from diagnosis to the occurrence of locoregional recurrence, while DMFS was the time from diagnosis to the occurrence of distant metastasis. DFS was defined as the time from diagnosis to the occurrence of locoregional relapse, distant metastasis, or death from any cause, whichever occurred first. OS duration was determined as the time from diagnosis to the date of death from any cause or the last recorded date when the patient was known to be alive.

### Statistical analysis

The differences in patient characteristics between the two treatment arms were compared using the chi-square test or Fisher’s exact test. Multivariate logistic regression analysis was used to determine the independent factors influencing the CR rate after induction therapy. The survival curves were plotted using the Kaplan-Meier method. Statistical analysis in this study was conducted using the IBM SPSS 26.0 software package (IBM Corp., Armonk, NY), with a significance level of P <0.05 indicating statistical significance.

## Results

### Patient characteristic

A total of 225 patients were included in this study (**Table 1**). Among these patients, 166 (72.5%) were male, 204 (90.7%) were WHO type III subtype, 163 (72.4%) were at T3–4 stage, 175 (77.8%) were at N2–3 stage, and 201 (89.3%) had detectable EBV-DNA before treatment. Regarding induction treatment, 43 (19.1%), 119 (52.9%), and 63 (28.0%) received GP, TP, and TPF regimens, respectively. There were 193 (85.8%) patients treated with IC alone and 32 (14.2%) patients received IC + PD-1 inhibitor. Of those receiving PD-1 inhibitor, 17 (53.1%) received Camrelizumab, and 15 (46.9%) received Tislelizumab. There were no significant differences in age, gender, smoking history, alcohol history, T stage, N stage, histological subtype, and EBV-DNA levels between patients receiving IC and IC + PD-1 inhibitor (**Table 1**).

**Table 1:** Patient characteristics between those treated with induction chemotherapy alone and induction chemotherapy plus PD-1 inhibitor.

Variables	n	IC	IC + PD-1 inhibitor	P
Age (years)				
<50	127	108 (56.0)	19 (59.4)	0.718
≥50	98	85 (44.0)	13 (40.6)	
Gender				
Male	59	53 (27.5)	6 (18.8)	0.299
Female	166	140 (72.5)	26 (81.3)	
Smoking history				
No	115	102 (52.8)	13 (40.6)	0.200
Yes	110	91 (47.2)	19 (59.4)	
Alcohol history				
No	148	121 (62.7)	27 (84.4)	0.017
Yes	77	72 (37.3)	5 (15.6)	
Histology				
WHO II	21	20 (10.4)	1 (3.1)	0.324
WHO III	204	173 (89.6)	31 (96.9)	
T stage				
T1	24	22 (11.4)	2 (6.3)	0.674
T2	38	34 (17.6)	4 (12.5)	
T3	117	98 (50.8)	19 (59.4)	
T4	46	39 (20.2)	7 (21.9)	
N stage				
N0	1	0 (0.0)	1 (3.1)	0.100
N1	49	43 (22.3)	6 (18.8)	
N2	87	74 (38.3)	13 (40.6)	
N3	88	76 (39.4)	12 (37.5)	
AJCC stage				
III	104	88 (45.6)	16 (50.0)	0.644
IVA	121	105 (54.4)	16 (50.0)	
Pretreatment EBV-DNA level				
Undetected	24	21 (10.9)	3 (9.4)	1.000
Detective	201	172 (89.1)	29 (90.6)	

IC, induction chemotherapy; WHO, World Health Organization; T, tumor; N, nodal; AJCC, American Joint Committee on Cancer; EBV-DNA, Epstein Barr virus-deoxyribonucleic acid.

### Treatment response

**Figure 1:**
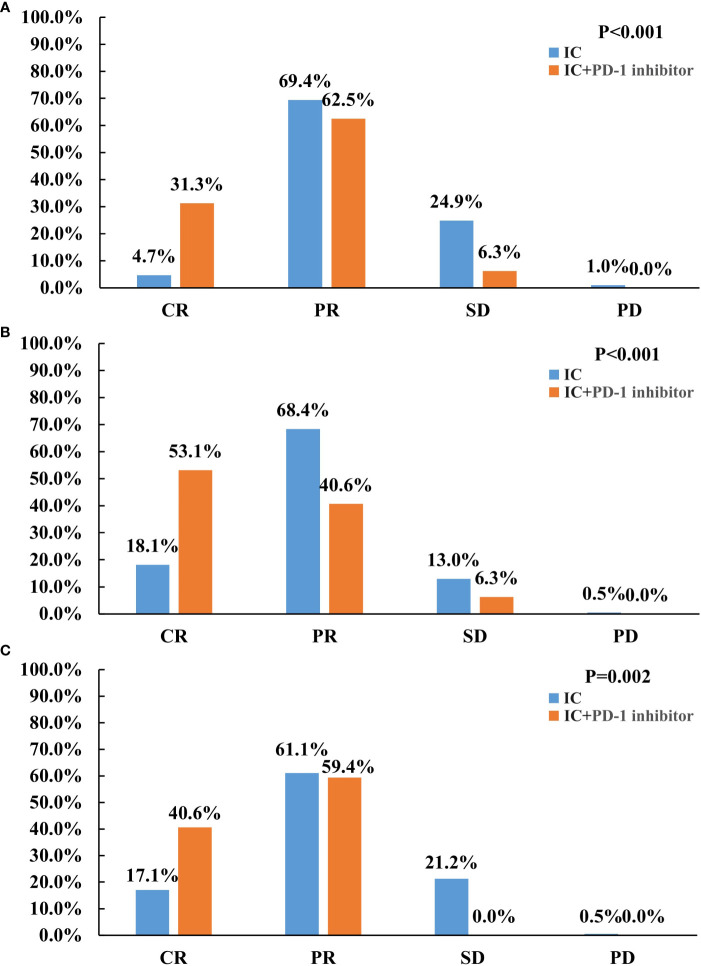
Treatment response between induction chemotherapy alone and induction chemotherapy plus PD-1 inhibitor in the entire cohort **(A)**, primary nasopharyngeal tumors **(B)**, and metastatic cervical lymph nodes **(C)**.

**Figure 2:**
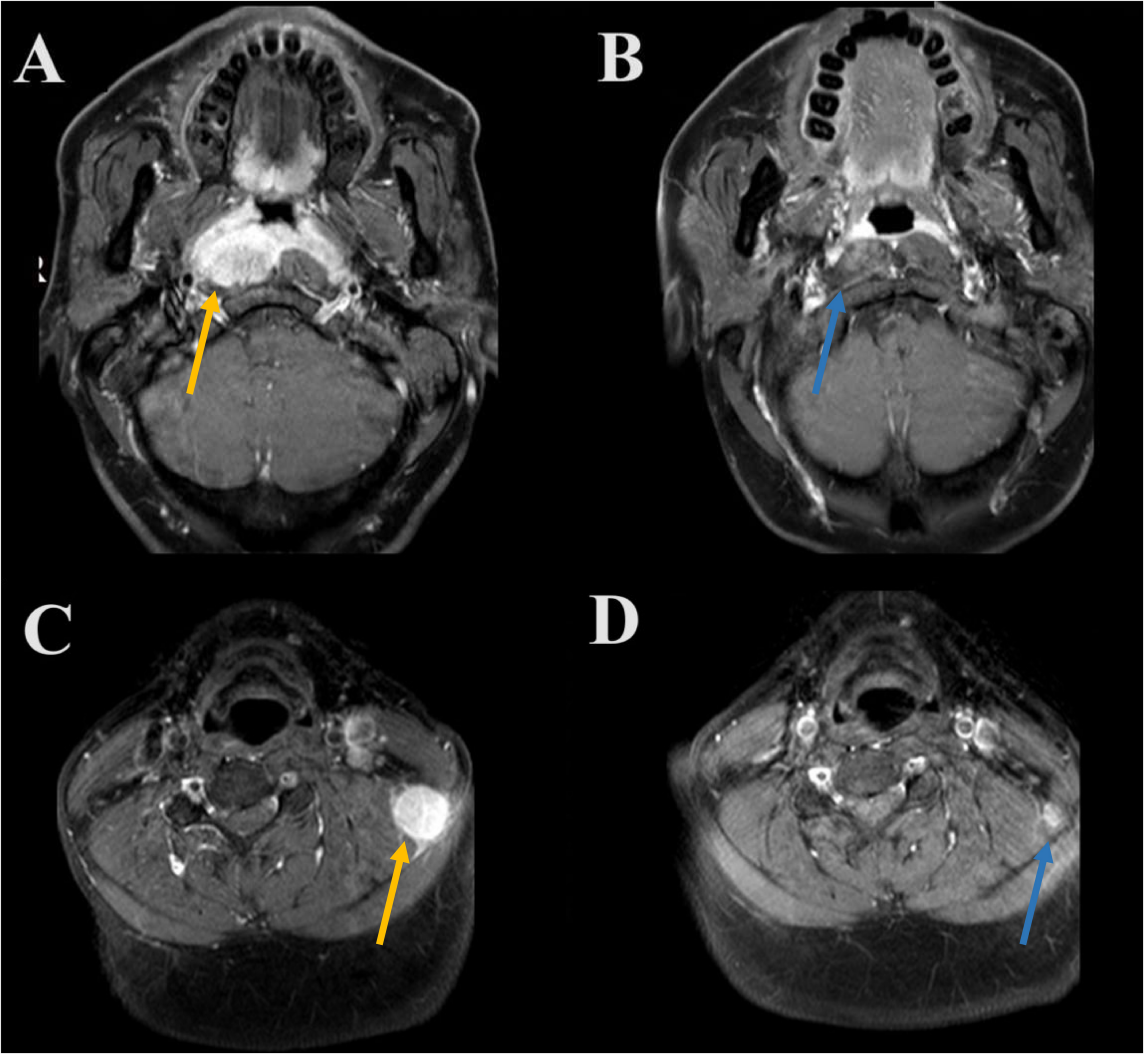
A patient who achieved complete response after induction chemotherapy plus PD-1 inhibitor (**A**, primary nasopharyngeal tumors before induction therapy [orange arrow]; **B**, complete response to primary nasopharyngeal tumors after induction therapy [orange arrow]; **C**, metastatic cervical lymph node before induction therapy [blue arrow]; **D**, complete response to metastatic cervical lymph node after induction therapy [blue arrow]).

All patients underwent efficacy evaluation after induction therapy (**Figure 1A**). The addition of PD-1 inhibitor to IC significantly improved the tumor response than those receiving IC alone. The CR, PR, SD, and PD rates of 4.7%, 69.4%, 24.9%, and 1.0%, respectively, for those treated with IC alone. In those with IC + PD-1 inhibitor, the CR, PR, SD, and PD rates were 31.3%, 62.5%, 6.3%, and 0%, respectively (P<0.001). There was a significant difference in ORR between those treated with IC alone and IC + PD-1 inhibitor (74.1% vs. 93.8%, P=0.012). **Figure 2** shows a patient who achieved CR after IC + PD-1 inhibitor.

We also evaluated the primary nasopharyngeal tumors and metastatic cervical lymph nodes separately. The addition of PD-1 inhibitor to IC also significantly improved the response to the primary nasopharyngeal tumors (P<0.001) (**Figure 1B**) and metastatic cervical lymph nodes (P=0.002) (**Figure 1C**), respectively. Regarding ORR, IC + PD-1 inhibitor could improve the ORR of metastatic cervical lymph nodes than those treated with IC alone (P=0.001), but there was a similar ORR for primary nasopharyngeal tumors between the treatment arms (P=0.386).

### Predictive factors associated with complete response after induction therapy

We conducted a multivariate logistic regression analysis to assess the independent predictors influencing the CR rate of patients after induction therapy (**Table 2**). The results revealed that receiving PD-1 inhibitor was an independent predictor influencing the CR rate of patients (odds ratio [OR] 9.814, 95% confidence interval [CI] 3.464–27.804, P<0.001). We also found that the CR rate of stage III patients was significantly higher than that of stage IVA patients (OR 3.886, 95% CI 1.269–11.906, P=0.017). The sensitivity analyses also showed that the additional PD-1 inhibitor to IC was the independent predictor influencing the CR of the primary nasopharyngeal tumors (OR 5.378, 95% CI 2.413–11.989, P<0.001) (**Table 3**) and metastatic cervical lymph nodes (OR 3.317, 95% CI 1.492–7.374, P=0.003) (**Table 4**).

**Table 2:** Multivariate logistic regression analysis for independent predictors influencing the complete response rate of the entire cohort.

Variables	OR	95%CI	P
Age (year)			
<50	1		
≥50	1.014	0.327–3.147	0.981
Gender			
Male	1		
Female	1.446	0.323–6.484	0.630
Smoking history			
No	1		
Yes	1.352	0.370–4.943	0.649
Alcohol history			
No	1		
Yes	0.245	0.048–1.249	0.091
Histology			
WHO II	1		
WHO III	2.631	0.615–11.301	0.998
AJCC stage			
III	1		
IVA	3.886	1.269–11.906	0.017
Pretreatment EBV DNA level			
Undetected	1		
Detective	1.112	0.125–9.909	0.924
PD-1 inhibitor			
No	1		
Yes	9.814	3.464–27.804	<0.001

IC, induction chemotherapy; WHO, World Health Organization; AJCC, American Joint Committee on Cancer; EBV-DNA, Epstein Barr virus-deoxyribonucleic acid; OR, odds ratio; CI, confidence interval.

**Table 3:** Multivariate logistic regression analysis for independent predictors influencing the complete response rate to primary nasopharyngeal tumors.

Variables	OR	95%CI	P
Age (years)			
<50	1		
≥50	1.850	0.947–3.617	0.072
Gender			
Male	1		
Female	1.100	0.409–2.956	0.851
Smoking history			
No	1		
Yes	1.220	0.520–2.859	0.648
Alcohol history			
No	1		
Yes	0.874	0.397–1.922	0.737
Histology			
WHO II	1		
WHO III	2.899	0.621–13.532	0.176
AJCC stage			
III	1		
IVA	1.817	0.932–3.544	0.080
Pretreatment EBV DNA level			
Undetected	1		
Detective	1.006	0.333–3.038	0.991
PD-1 inhibitor			
No	1		
Yes	5.378	2.413–11.989	<0.001

IC, induction chemotherapy; WHO, World Health Organization; AJCC, American Joint Committee on Cancer; EBV-DNA, Epstein Barr virus-deoxyribonucleic acid; OR, odds ratio; CI, confidence interval.

**Table 4:** Multivariate logistic regression analysis for independent predictors influencing the complete response rate to metastatic cervical lymph nodes.

Variables	OR	95%CI	P
Age (years)			
<50	1		
≥50	0.913	0.449–1.856	0.801
Gender			
Male	1		
Female	2.028	0.732–5.615	0.174
Smoking history			
No	1		
Yes	0.928	0.403–2.138	0.862
Alcohol history			
No	1		
Yes	0.75	0.339–1.661	0.479
Histology			
WHO II	1		
WHO III	0.545	0.187–1.593	0.268
AJCC stage			
III	1		
IVA	1.629	0.823–3.225	0.161
Pretreatment EBV DNA level			
Undetected	1		
Detective	0.711	0.248–2.034	0.525
PD-1 inhibitor			
No	1		
Yes	3.317	1.492–7.374	0.003

IC, induction chemotherapy; WHO, World Health Organization; AJCC, American Joint Committee on Cancer; EBV-DNA, Epstein Barr virus-deoxyribonucleic acid; OR, odds ratio; CI, confidence interval.

### Toxicity

**Figure 3:**
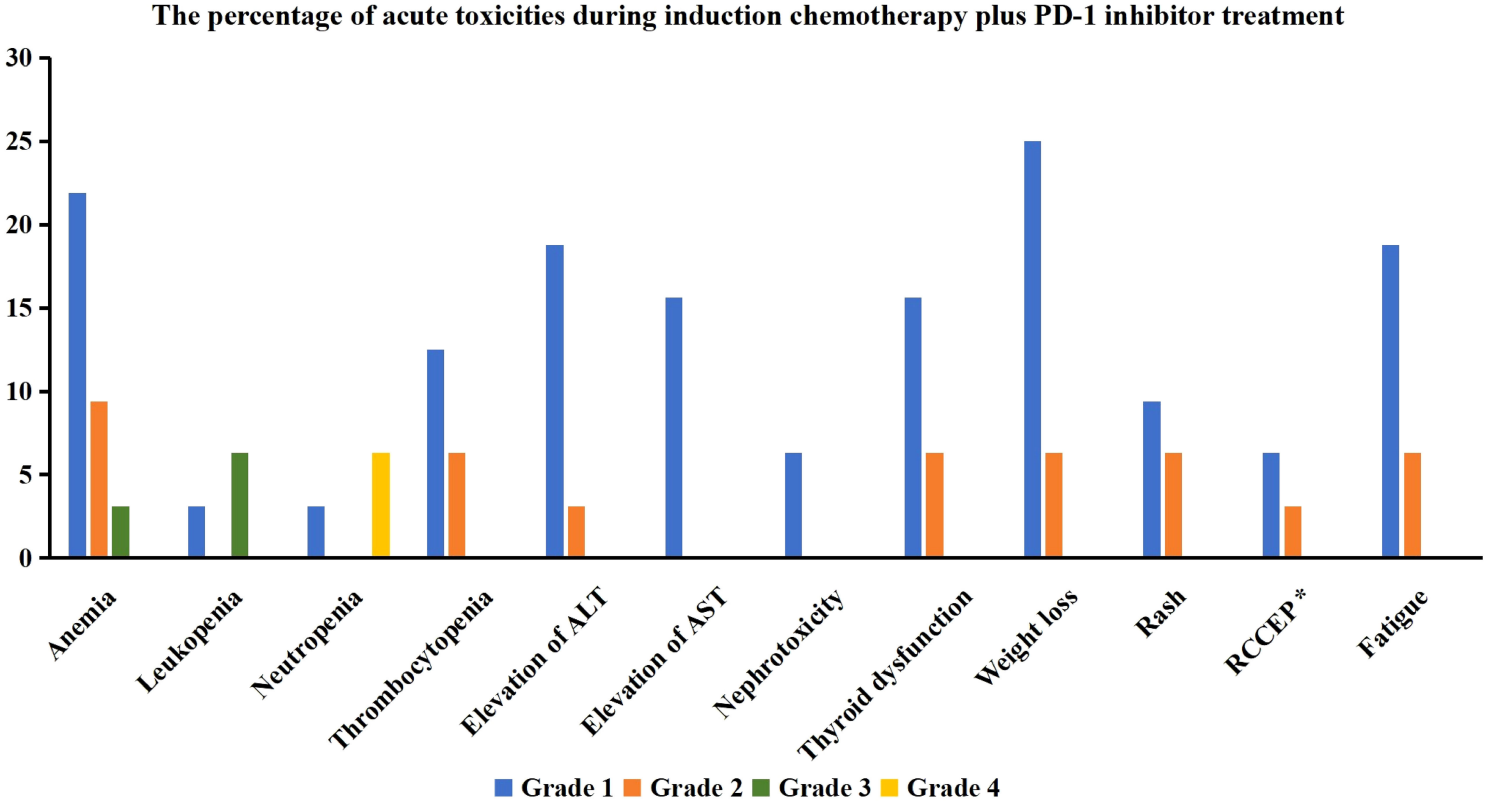
The percentage of acute toxicities during induction chemotherapy plus PD-1 inhibitor treatment (ALT, alanine aminotransferase; AST, aspartate aminotransferase; RCCEP, reactive cutaneous capillary endothelial proliferation) (*17 patients received treatment with Camrelizumab).

We assessed the treatment toxicities in those receiving PD-1 inhibitor (n=32) (**Figure 3**). Regarding hematological toxicity, 11 (34.4%), 6 (18.8%), 3 (9.4%), and 3 (9.4%) patients experienced anemia, thrombocytopenia, leukopenia, and neutropenia, respectively. Five patients (15.7%) experienced grade 3–4 hematological toxicity, but all returned to normal after symptomatic treatment.

In terms of non-hematological toxicity, 7 (21.9%), 7 (21.9%), 5 (15.6%), and 2 (6.3%) patients experienced alanine aminotransferase elevation, thyroid dysfunction, aspartate aminotransferase elevation, and creatinine elevation, respectively, all of which were grade 1 or 2 toxicities without any grade 3 or higher toxicities were found.

Among the 17 patients who received treatment with Camrelizumab, three patients (17.6%) experienced reactive cutaneous capillary endothelial proliferation, all of which were grade 1 or 2. One patient (3.1%) developed scattered rashes during treatment but improved after symptomatic treatment.

### Short-term survival

**Figure 4:**
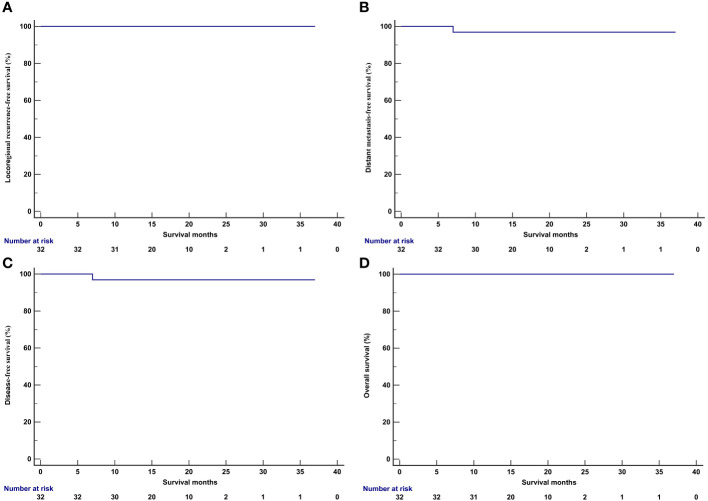
Kaplan-Meier curve of locoregional recurrence-free survival **(A)**, distant metastasis-free survival **(B)**, disease-free survival **(C)**, and overall survival **(D)** in patients receiving induction chemotherapy plus PD-1 inhibitor.

In those treated with IC and PD-1 inhibitor, all patients received and completed the recommended radiotherapy and concurrent chemotherapy. The median follow-up period was 17.0 months (range, 9–37 months). One patient experienced an elevation of plasma EBV-DNA levels 7.3 months after NPC diagnosis, and PET/CT confirmed thoracic vertebral metastasis. The patient received a combination of Tislelizumab and the GP regimen. After two cycles of treatment, EBV-DNA became undetectable, and PET/CT showed no metabolic activity in the thoracic vertebral metastases. At the time of data publication, the patient had completed the fifth cycle of treatment. Another patient was diagnosed with primary hepatocellular carcinoma 17.9 months after NPC diagnosis and underwent surgical treatment. None of the patients experienced locoregional recurrence or death during the follow-up period. The one-year LRFS, DMFS, DFS, and OS were 100%, 96.9%, 96.9%, and 100%, respectively (**Figure 4**).

## Discussion

Currently, the recommendation for PD-1 inhibitor in NPC mainly focuses on recurrent or metastatic patients and shows improved tumor response rates and survival rates compared to those treated with chemotherapy alone (9–12). Theoretically, integrating PD-1 inhibitor into the induction therapy may achieve a better response for LANPC compared to those treated with IC alone. To test this hypothesis, we explored the clinical response and toxicity of IC + PD-1 inhibitor in patients with LANPC. We found that adding PD-1 inhibitor to IC significantly improved the CR of patients while maintaining acceptable treatment toxicities.

The response to IC is closely associated with the survival of patients. Results from the secondary analysis of the prospective study have shown that patients achieving a CR after IC with a GP regimen have significantly better OS than those with PR or SD/PD, with 5-year OS of 100%, 88.4%, and 61.5% respectively (P=0.005) (5). Achieving a CR is crucial in cancer treatment as it indicates the eradication of visible tumor cells and is associated with improved long-term outcomes. However, it should be noted that in prospective studies, the CR rates after GP, TPF, and TP regimen were only 10% (5), 11.3% (8), and 2.8% (7), respectively. Several retrospective studies, including ours, have also found that the CR rate after IC did not exceed 5% (6, 16). In this study, the CR rate was only 4.8% in patients receiving IC, which was consistent with the findings of the above studies. However, when we added PD-1 inhibitor to IC, the CR rate reached 34.4%, and the ORR after induction therapy reached 93.8%. In two prospective studies combining the GP regimen with Tislelizumab, the overall CR rate was 41.3–50.0%, and the ORR was 88.9% to 95.8% (17, 18). Based on our results and the findings of the above prospective studies, integrating PD-1 inhibitor into induction therapy may improve the CR rate and potentially have an impact on survival outcomes.

Our study further showed that the CR rate was 56.3% and 43.8% in the primary nasopharyngeal tumors and metastatic cervical lymph nodes after IC + PD-1 inhibitor, respectively. Furthermore, adding PD-1 inhibitor was identified as an independent predictive factor affecting the CR rate of patients. Currently, there is still a lack of separate evaluations of responses to IC + PD-1 inhibitor in prospective studies. A retrospective study by Xiang et al. found that adding PD-1 inhibitor to IC significantly improved the CR rate of the primary nasopharyngeal tumors (0.8% vs. 14%, P<0.001) and metastatic cervical lymph nodes (22.3% vs. 36.8%, P=0.021) compared to IC alone. However, for patients receiving IC with the GP regimen, adding PD-1 inhibitor only improved the CR rate of the primary nasopharyngeal tumors (17.0% vs. 1.5%, P=0.002), while it did not affect the CR rate of metastatic cervical lymph nodes (31.9% vs. 27.3%, P=0.400) (19). In our previous study, we found that patients achieving CR in the primary nasopharyngeal tumors or metastatic cervical lymph nodes after IC had better progression-free survival (6). Therefore, in the era of immunotherapy, more studies are required to assess the effect of treatment response to IC + PD-1 inhibitor on survival outcomes in LANPC.

Several prospective studies on head and neck cancer have found that adding PD-1/PD-L1 inhibitor (Pembrolizumab or Avelumab) to CCRT and subsequent maintenance PD-1/PD-L1 inhibitor for one year did not significantly improve survival outcomes compared to those treated with CCRT alone (20, 21). However, patients enrolled in both studies did not receive induction therapy, therefore the response to IC and PD-1 inhibitor could not be evaluated. Since cervical lymph nodes are a standard target volume for radiotherapy of the NPC, radiation to the lymph node drainage area may impair the immune response caused by PD-1 antibodies. Therefore, initiating PD-1 inhibitor treatment before radiotherapy may better activate the immune system and enhance the efficacy of immunotherapy. For LANPC patients, initial findings from the CONTINUUM study suggested that the use of Sintilimab (PD-1 inhibitor) in combination with IC, CCRT, and adjuvant therapy significantly improves the 3-year event-free survival rate compared to those treated with IC plus CCRT (86.1% vs. 76.0%) (22). In our study, we found short-term survival was excellent by using IC and PD-1 inhibitor with a median follow-up time of 17.0 months. Several studies have shown that chemotherapy could induce antigen presentation and induce expression of immune checkpoints (23). In addition, chemotherapeutic agents-induced immunogenic cell death and their immune stimulation activity are considered the main mechanisms of combination therapy (24). Therefore, early initiation of immunotherapy may be beneficial in LANPC.

In this study, 53.1% of patients received treatment with Camrelizumab, and 46.9% received treatment with Tislelizumab. Multivariate analysis showed no significant difference in the CR rate between the two PD-1 inhibitors. Li et al. found that IC with GP regimen had an immune modulation effect in LANPC and did not weaken the cytotoxic activity and proliferative capacity of T cells (25). In addition, the effects of nab-paclitaxel on modulation of the cancer-immunity cycle provide potential avenues for a combined therapeutic rationale to improve the efficacy of PD-1 inhibitor (26). Several studies on lung and breast cancer have shown that the combination of nab-paclitaxel and PD-1 inhibitor results in significantly better survival than nab-paclitaxel alone (27, 28). The use of corticosteroids in immunotherapy may affect the efficacy of PD-1 inhibitors, while paclitaxel and docetaxel often require corticosteroid pretreatment. Therefore, the combination of nab-paclitaxel and PD-1 inhibitor may have advantages. Currently, we are conducting a prospective Phase II study to explore the impact of the TPF regimen based on nab-paclitaxel combined with Camrelizumab on tumor response rate and survival in patients with LANPC.

In this study, the common toxicities were hematological toxicity and liver function damage, which were similar to the common toxicities of chemotherapy. Thyroid dysfunction is a common toxicity to PD-1 inhibitor, especially hypothyroidism (29, 30). In this study, we observed that 21.9% of patients experienced hyperthyroidism during IC and PD-1 inhibitor, and no cases of hypothyroidism were observed. The study by Zhang et al. also found that out of 25 patients, 9 (36%) had thyroid dysfunction, with 8 patients having hyperthyroidism and 1 patient having hypothyroidism (18). Studies on NPC and lung cancer have found that patients who experience thyroid dysfunction during PD-1 inhibitor treatment have better disease control rates (29, 30). However, due to the small sample size, we did not observe the correlation between thyroid dysfunction and the CR rate of patients. In the future, more samples need to be accumulated to explore the correlation between thyroid dysfunction and the efficacy of treatment in patients.

We needed to acknowledge several limitations of our study. First, the combination of IC and PD-1 inhibitor has not yet been approved for LANPC, and our study only included a small sample size. Second, the recording of toxicities during treatment may be insufficient due to the retrospective analysis. Therefore, we did not compare the differences in adverse reactions between IC and IC combined with PD-1 inhibitor. However, based on the results from the prospective randomized controlled studies (10–12), the incidence of all adverse events, grade 3 or greater adverse events, and fatal adverse events were similar between those treated with GP and GP + PD-1 inhibitor in recurrent or metastatic NPC. Third, the immune infiltration characteristics such as PD-L1 expression were not routinely assessed in patients with LANPC in our institution, thus we were unable to evaluate the relationship between the CR rate and PD-L1 expression status. However, several prospective randomized controlled studies in recurrent or metastatic NPC have found no significant correlation between baseline PD-L1 expression and objective response rate or progression-free survival (10–12). Finally, our study has a relatively short follow-up time, and a longer follow-up is needed to clarify the impact of a combination of IC and PD-1 inhibitor on the survival of patients.

## Conclusions

In conclusion, the addition of PD-1 inhibitor to IC has promise as an effective treatment approach for LANPC. More studies are expected to provide further insights into the optimal use of this treatment strategy, paving the way for more personalized and effective treatment options for patients with LANPC.

## Data availability statement

The raw data supporting the conclusions of this article will be made available by the authors, without undue reservation.

## Ethics statement

The studies involving humans were approved by The First Affiliated Hospital of Xiamen University. The studies were conducted in accordance with the local legislation and institutional requirements. The participants provided their written informed consent to participate in this study.

## Author contributions

Y-FY: Writing – original draft, Visualization, Validation, Supervision, Software, Resources, Project administration, Methodology, Investigation, Funding acquisition, Formal analysis, Data curation, Conceptualization. G-ZL: Writing – original draft, Visualization, Validation, Supervision, Software, Resources, Project administration, Methodology, Investigation, Funding acquisition, Formal analysis, Data curation, Conceptualization. R-JW: Writing – review & editing, Visualization, Validation, Supervision, Software, Resources, Project administration, Methodology, Investigation, Funding acquisition, Formal analysis, Data curation, Conceptualization. Y-KS: Writing – review & editing, Visualization, Validation, Supervision, Software, Resources, Project administration, Methodology, Investigation, Funding acquisition, Formal analysis, Data curation, Conceptualization. S-GW: Writing – review & editing, Visualization, Validation, Supervision, Software, Resources, Project administration, Methodology, Investigation, Funding acquisition, Formal analysis, Data curation, Conceptualization.
